# Catalytic Gas-Phase Glycerol Processing over SiO_2_-, Cu-, Ni- and Fe- Supported Au Nanoparticles

**DOI:** 10.1371/journal.pone.0142668

**Published:** 2015-11-18

**Authors:** Maciej Kapkowski, Tomasz Siudyga, Rafal Sitko, Józef Lelątko, Jacek Szade, Katarzyna Balin, Joanna Klimontko, Piotr Bartczak, Jaroslaw Polanski

**Affiliations:** 1 Institute of Chemistry, University of Silesia, Szkolna 9, 40–006 Katowice, Poland; 2 Department of Chemistry, Silesian University of Technology, 44–100 Gliwice, Poland; 3 Institute of Materials Science, University of Silesia, 75 Pułku Piechoty 1A, 41–500 Chorzów, Poland; 4 A.Chełkowski Institute of Physics, University of Silesia, Silesian Center for Education and Interdisciplinary Research 41–500 Chorzów, Poland; Queen's University Belfast, UNITED KINGDOM

## Abstract

In this study, we investigated different metal pairings of Au nanoparticles (NPs) as potential catalysts for glycerol dehydration for the first time. All of the systems preferred the formation of hydroxyacetone (HYNE). Although the bimetallics that were tested, i.e., Au NPs supported on Ni, Fe and Cu appeared to be more active than the Au/SiO_2_ system, only Cu supported Au NPs gave high conversion (ca. 63%) and selectivity (ca. 70%) to HYNE.

## Introduction

Renewable naturally sourced carbohydrates, amino acids and triglycerides are available in vast quantities in our environment. This biomass, which is a product of living organisms, could be used as valuable feedstock for chemical processing; however, we need *novel chemistry to transform large amounts selectively and efficiently in their natural state without extensive functionalization and protection* [1]. For this reason, biomass conversion has received increasing attention in contemporary chemistry. Glycerol, a byproduct in biodiesel production, is one of the most widely available biosourced chemicals, making it an attractive target of investigations. Nanocatalysis is an interesting option in this area.

Glycerol oxidation in the liquid phase is probably the broadest processing opportunity that is catalyzed by Au NPs [2,3]. This reaction has been exhaustively studied recently to identify possible oxidates that can be connected by a complex reaction network [4–6]. The reaction usually requires the dilution of glycerol; however, the highly viscous liquid phase can also be efficiently oxidated on bimetallic Cu- and Ni-supported Au NPs. [7]. Gas-phase glycerol processing, which could be an alternative to liquid-phase reactions, allowed one to significantly decrease the viscosity of glycerol. In fact, this has recently been tested as a possible reaction to obtain acrolein. In particular, mixed Nb–W-oxide catalyzes this conversion yielding more than 70% acrolein with selectivity above 40%. Even better selectivity was obtained using a catalyst with tungsten oxide supported on titania, which gave 80% selectivity under aerobic conditions at a high conversion [8–11].

Nanocatalysts are extremely sensitive toward structure differentiation and their activity and selectivity depend not only on the nano-metal and support type but also on the size, shape and composition [12]. Thus, the optimization of such materials is an open issue. In fact, more efficient catalysts are still being sought in order to run reactions that have higher yields and higher selectivity under mild conditions. Other important problems to be addressed include reducing the fraction of noble metals, facilitating the separation of the catalyst, improving reusability and reducing the contamination of the final products. Gold nanoparticles (Au NPs) catalyze a variety of reactions [13–16]. As they tend to agglomerate, they are usually supported on carriers to form more stable catalytic systems. Generally, Au NPs are available on a variety of supports ranging from carbon-like graphite to inorganic materials. Au NPs have also been supported on a variety of metallic grains and have provided highly efficient catalytic systems [17].

In particular, we have recently shown that bimetallic catalysts that are constructed of Au or Pd NPs if immobilized in the debris of silica supported on metallic carriers can provide highly efficient catalysts for a variety of reactions [7,18,19]. In turn, a variety of nano-Au/C supported catalytic systems have been developed recently that are efficient for glycerol oxidation [7,20]. Additionally, various mixed oxide catalysts were used in glycerol oxydehydration in the gas phase [11]. Recent developments in this field were reviewed in the reference [21]. In this study, we tested the reaction paths and the efficiency of glycerol dehydration in the gas phase, which if previously investigated on Al_2_O_3_-, SiO_2_- and TiO_2_-supported Nb- and W-oxide catalysts during a long 3-h operation gave glycerol conversions of ca. 40% and a selectivity to acrolein of ca. 70% [8]. Here, we tested the activity of Au NPs that were supported on SiO_2_-, Cu-, Ni- and Fe in a flow reactor while reactants were in short-time contact with the catalysts. We carefully investigated the reaction paths, glycerol conversion and product selectivity in order to determine the best possible catalyst options and reaction conditions for the system.

## Material and Methods

### A. Preparation of Au NPs on sol-gel silica and bimetallic Au NPs on Cu-, Ni-, Fe- carriers

The Au/silica nanocatalyst was prepared according to a procedure that was optimized. The intermediate carrier, amorphous SiO_2,_ was prepared using the sol-gel technique (Stöber method) with tetraethyl orthosilicate (TEOS) [22,23]. In the general procedure, a solution of anhydrous ethanol (800 mL) and aqueous ammonia (25 wt.%, 135 mL) was stirred for 15 min, followed by the addition of tetraethyl orthosilicate (60 mL). The reaction mixture was vigorously stirred for 3 h at room temperature, thereby affording a colloidal silica suspension (with a theoretical mass of silica of 16.1 g). The colloidal silica suspension that was obtained was centrifuged, washed to neutral pH (deionized water) and suspended in deionized water (20 mL) in an ultrasound bath and stirred for 90 min. A solution containing a gold precursor (1.41 g 30% chloroauric acid for the preparation of 1.5% Au/SiO_2_) in deionized water (10 mL) was added dropwise into the carrier that was obtained, i.e. colloidal silica and mixed in an ultrasound bath for 30 min. Next, it was dried to a constant weight at 60–90°C for about 12 h in the dark, ground and sieved. The reduction was conducted in an oven under hydrogen at 500°C for 4 h.

Bimetallic Au catalysts were prepared using an approach which includes several steps. The nanoparticles were transfered from the intermediate carrier, i.e. SiO_2,_ to the target carrier by the selective digestion of silica using an NaOH solution, washed to neutral pH, dried after which the nanomaterial that was obtained was sieved. In general, 9.9 parts of powder, i.e. Ni, Fe or Cu, with a grain size below 100 μm and 6.67 parts of powder containing 1.5% of the gold NPs on the amorphous silica were treated in mechanical stirrer and ultrasonic cleaner for 10 minutes in a mixture of water and ethanol (9:1). While stirring constantly 200 parts of 40% aqueous NaOH solution was added to the suspension after which the stirring was continued for 4 hours at 80°C, whereupon the suspension was allowed to stand for about 18 h until the suspended solids sedimented. The suspension was centrifuged and the supernatant was decanted, the precipitate was washed five times with deionized water and centrifuged again to achieve neutral pH of the supernatant. The precipitate was washed with deionized water, centrifuged and the supernatant was removed. The product that was obtained contained 1.0% gold with a grain size of less than 10 nm.

The resulting preparations of silica and bimetallic catalysts were examined using the SEM, TEM, HRTEM and EDXRF techniques. The samples of the catalysts were suspended in ethanol, sonicated for 15 min and the resulting materials were deposited on carbon adhesive tape for the preparation of the samples for TEM analyses. The transmission electron microscopy (TEM) images of the resulting composites were obtained using a JEOL 2000 FX operating at 200 kV or high resolution (HRTEM) JEM 3010 microscopes (both with EDS systems for the microanalysis of the chemical composition). Scanning electron microscopy (SEM) using a JSM6480 or a PHILIPS XL 30 was used to investigate the morphology of the composite powders.

The chemical analysis was performed using an energy-dispersive X-ray fluorescence (EDXRF) spectrometer–Epsilon 3 (Panalytical, Almelo, The Netherlands) with a Rh target X-ray tube operating at the max. voltage of 30 keV and max. power of 9W. The spectrometer is equipped with a thermoelectrically cooled silicon drift detector (SDD) with an 8 μm Be window and a resolution of 135 eV at 5.9 keV. The quantitative analysis, which was based on fundamental parameter method and following measurement conditions: 5 kV, 300 s counting time, helium atmosphere for Si, Al and P determination; 12 kV, 300 s counting time, helium atmosphere, 50 μm Al primary beam filter for Ca, Ti and V; 20 kV, 120 s counting time, air atmosphere, 200 μm Al primary beam filter for Cr, Mn and Fe; and 30 kV, 120 s counting time, air atmosphere, 100 μm Ag primary beam filter for Au, Ni, Cu, Zn and Br, was performed using Omnian software. The current of the X-ray tube was fixed so that it did not exceed a dead-time loss of ca. 50%.

We used 3Flex Surface Characterization Analyzer (Micromeritics, USA) to determine the N_2_ adsorption isotherm at 77 K in the range of 0.05 to 0.3 relative pressure in order to calculate the BET surface area. Prior to the measurement, the sample was degassed in a vacuum at 350°C for 5 h.

The electronic structure was studied in films with the use of Prevac/VGScienta photoelectron spectrometer. Monochromatic Al*K*_α_ x-ray radiation (h*ν* = 1486.7 eV) was used to obtain the photoelectron spectra of core levels of particular elements. The chemical composition and analysis of the structure of the obtained XPS multiplets was performed with the use of Multipak programme from Physical Electronics.

The X-ray diffraction measurements were carried out using a high-resolution Siemens diffractometer (θ-θ) D 5000 with filtered CuK_α_ radiation (40kV, 30mA). Qualitative phase analysis employed the “Diffract AT Search/Match Program” and the data from the International Centre for Diffraction Data (JCPDS-ICDD).

### B. Glycerol dehydration

Glycerol (0.6–1.5 mL, 1.0–13.6 mol/L; Fisher BioReagents^®^: glycerol for molecular biology) dehydration was performed under atmospheric pressure in a quartz flow microreactor with a fixed nano-Au catalyst bed (200 mg, 10.0–15.0 μmol Au) with a diameter of 7.5 mm. Glycerol was continuously introduced into the evaporation system (flow rate of 0,3 cm^3^/min.; 11330 kg/h kg_met_ in relation to the Au NPs) and glycerol vapors with inert gas N_2_ (the flow rate of 2 dm^3^/h) were continuously injected into the microreactor.

The products that resulted from the glycerol processing were collected and analyzed using spectroscopic techniques. In particular, 0.01 mL of the sample was dissolved in 0.6 mL of deuterium oxide and analyzed using the ^1^H and ^13^C NMR techniques. Additionally, the 2D COSY and HMQC methods were used to identify and quantify the products. The spectra were recorded on Bruker Avance 400 or 500 spectrometers with TMS as the internal standards (400 MHz, ^1^H, 101 MHz ^13^C or 500 MHz, ^1^H, 126 MHz ^13^C) at room temperature. The signal from water was suppressed using 90 water-selective pulses (zggpwg). The results had an error of ±2% throughout the experiments. The formation of 1-hydroxyacetone was confirmed using spectroscopic techniques. **Eqs. (1)–(3)** were used to calculate conversion, product selectivity and yield, respectively. The precise mass balance of glycerol was kept during the experiment, i.e the total incoming glycerol was balanced by the products outcome through the catalyst bed.

$$Conversion\;(mol\%)\;=\;\frac{\left(initial\;moles\;of\;glicerol\;-\;final\;moles\;of\;glycerol\right)}{initial\;moles\;of\;glicerol}\;x\;100$$ (1)

$$Selectivity\;of\;products\;(mol\%)\;=\;\frac{percentage\;amount\;of\;product\;formed}{the\;total\;percentage\;of\;all\;product\;formed}\;x\;100$$ (2)

$$Yield\;(\%)\;=\;\frac{conversion\;of\;glycerol\;x\;selectivity\;of\;desired\;product}{100}$$ (3)

## Results and Discussion

### A. The characteristics and structure of the catalysts

Over the past few years, a number of techniques have been developed for the production of nanosized metallic particles and their distribution on different carriers. The methods that are currently being used, which are based on “the bottom-up” and “the top-down” techniques, still suffer from some disadvantages, including the broad-sized distribution of nanoparticles and their tendency to aggregate or form clumps [24,25]. To minimize these problems, we recently developed a novel method for the formation of bimetallic catalysts [18] in which we used amorphous silica that had been synthesized using the sol-gel [23] technique as the basic carrier. SEM observations indicated that the silica that was obtained using this method exhibited a regular spherical shape, a controlled size distribution and a uniform porous surface. This regular shape was preserved in Au NPs that were supported on the SiO_2_ carrier that was obtained using the Stöber method [22]. The Au/SiO_2_ system and final carrier were suspended in deionized water, placed in an ultrasound bath and stirred. Then, the SiO_2_ was digested. Silica-supported gold was prepared as was reported in reference [7].

**Fig 1:**
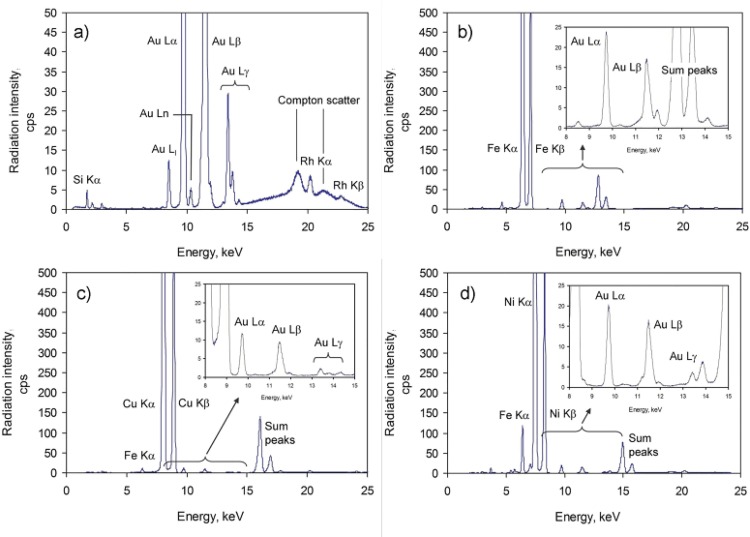
EDXRF spectra of Au/SiO_2_ (a), Au-Fe (b), Au/Cu (c) and Au/Ni(d) that were collected using an Rh target X-ray tube operated at 30kV and 300 μA.

**Fig 2:**
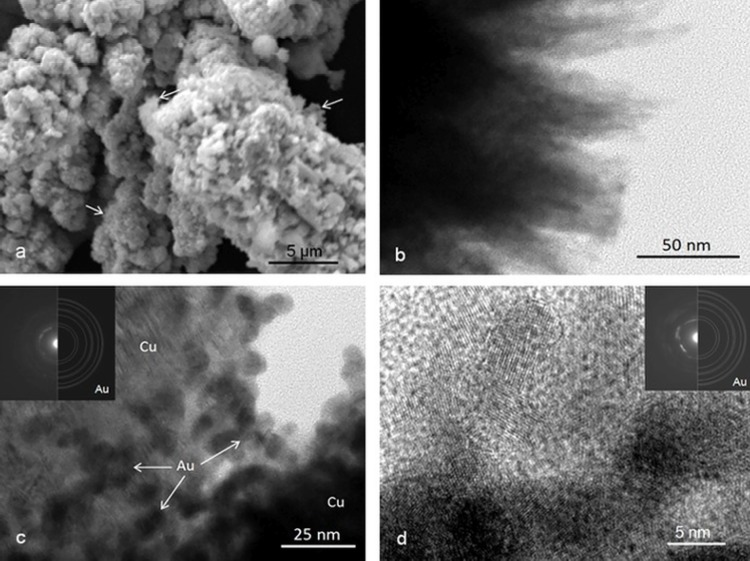
SEM (a) and TEM (b-d) images of 1.0% Au/Cu catalyst.

Herein, we additionally tested a previosuly used approach for other bimetallic systems, in particular, Cu-, Fe- and Ni-supported Au NPs. The 1.5% Au/SiO_2_ system was chosen as the intermediate carrier. We used the N_2_ adsorption isotherm to determine the specific surface area. The results are summarized in **Table 1.** The EDXRF spectra of Au/SiO_2_, Au/Fe, Au/Ni and Au/Cu catalysts **(Fig 1)** shows peaks of the matrix elements (Si Kα, Fe Kα, Fe Kβ, Cu Kα, Cu Kβ, Ni Kα, Ni Kβ at 1.74, 6.40, 7.06, 8.05, 8.90, 7.48, 8.26 keV, respectively) as well as several Au peaks that correspond to the L3 edge (Lα1, Lα2, Lβ2,15, Lβ5 and Lι at 9.71, 9.63, 11.57, 11.91, 8.49 keV), the L2 edge (Lβ1, Lγ1 and Lη at 11.44, 13.38, 10.31 keV) and the L1 edge (Lβ3, Lβ4, Lγ2 at 11.61, 11.21 and 13.71 keV). The spectra also reveal the presence of some minor and trace elements (Al, P, Ca, Ti, V, Cr, Mn, Zn and Br). **Table 2**presents the quantitative EDXRF analysis of the Au/SiO_2_, Au/Fe, Au/Ni and Au/Cu catalysts. The low porosity of Cu and Ni decides that, in comparison to SiO_2_, a larger fraction of Au NPs are available directly on the surfaces of these catalysts. SEM and TEM analyses proved that Au-Cu, Au-Fe or Au-Ni contact is formed in these catalysts **(Fig 2, S1 and S2 Figs).** The residual debris of the original Au/SiO_2_ conglomerates can still be detected on the surface of the metal **(Fig 2, S1 Fig).** Au content in a 1.0% Au/Ni catalyst was proved using the EDS method **(S1 Fig, S1 Table).** SEM investigations showed that the 1.0% Au/Cu system is a conglomerate of the highly developed surface **(Fig 2A).** The high degree of surface development was confirmed using TEM. **Fig 2B** shows the conglomerates that had a nanocrystalline texture. The phase of a low degree of crystallinity was also revealed **(arrows on Fig 2A and 2B).** The Au NPs were mainly in the form of conglomerates in which individual Au nanoparticles had an irregular oval shape with a 10.8 ± 2.2 nm diameter **(Fig 2C).** HRTEM images showed that in most cases these particles had a polycrystalline structure **(Fig 2D).**

**Table 1:** Specific surface area (SSA) of the tested catalysts.

Catalyst	SSA [m^2^/g]
1.5% Au/SiO_2_	266.10 ± 3.00
1.0% Au/Cu	93.70 ± 3.00
1.0% Au/Ni	116.32 ± 3.00
1.0% Au/Fe	141.90 ± 3.00

**Table 2:** Au NPs content as determined by EDXRF analysis over SiO_2-_ and Cu-, Ni- and Fe-carriers.

Chemical element^a^	Au content as determined by EDXRF [% m/m]			
	1.0% Au/Fe	1.0% Au/Ni	1.0% Au/Cu	1.5% Au/SiO_2_
Fe	98 ± 5.6	1.50 ± 0.096	-	0.0042 ± 0.00055
Ni	0.053 ± 0.0033	93 ± 4.4	-	0.0012 ± 0.00070
Cu	0.020 ± 0.0017	0.26 ± 0.010	98 ± 3.9	-
Si	0.31 ± 0.011	1.9 ± 0.13	0.063 ± 0.0082	97 ± 7.3
Au	1.02 ± 0.042	0.98 ± 0.050	1.13 ± 0.041	1.52 ± 0.12
Ca	0.13 ± 0.013	1.88 ± 0.095	0.069 ± 0.0057	-
Ti	0.11 ± 0.010	-	-	-
V	0.079 ± 0.0045	-	-	-
Br	0.052 ± 0.0030	-	-	-
P	-	0.24 ± 0.010	0.29 ± 0.023	-
Cr	-	0.15 ± 0.012	-	0.0019 ± 0.00033
Mn	-	0.038 ± 0.0028	-	-
Zn	-	0.061 ± 0.0043	-	-
Al_2_O_3_	-	-	-	0.41 ± 0.037
P_2_O_5_	-	-	-	0.54 ± 0.035
CaO	-	-	-	0.12 ± 0.012

a/ Na–below detection level.

The 1.0% Au/Ni catalyst was composed of irregular particles with smooth Ni surfaces **(area B in S1A Fig).** A higher content of Au was detected among these particles **(area A in S1A Fig).** This was analyzed further by TEM **(S1B Fig).** In the amorphous-nanocrystalline Si and Ni matrix, Au NPs were found both as individual particles that were distributed fairly apart (areas F, G) or as gathering more closely in the neighborhood areas (A, B). Monocrystalline particles of the Ni_2_Si intermetallic phase (area E) were also detected. The average Au NP had a diameter of 5.4 (±1.2) nm **(S1D Fig).**

The Au/Fe system has higher surface area than the other investigated ones **(S2A Fig).** Au NPs can be observed on the surface and chemical analysis (EDS) indicated 1.4 at. % Au, 0.4 at. % Ca and the traces of Al, Si i Mg. In Figure **(S2B and S2C Fig)** we show the microphotographs of this material, which indicated Au NPs. NP sizes are of the order of 10 nm **(S2D Fig).**

XPS analysis allowed to determine the chemical composition which is representative for the surface of the measured nanocomposites due to the limited electron mean free path of photoelectrons which can be estimated to about 3–4 nm. The relative ratio of Au with respect to supporting materials is different according to the XPS and EDXRF data **(S2 Table)**. For all systems the relative content is more rich in Au (XPS data) indicating to localization of the Au nanoparticles on the very surface of the supporting material. In turn, EDXRF results refer to the surface composition related of the deeper surface skin layer. The effect is most pronounced for Au/Ni and Au/Fe. For Au/SiO_2_ there is only a small difference in relation to EDXRF what can be related to the localization of Au nanoparticles in the pores of the SiO_2_ particles.

**Fig 3:**
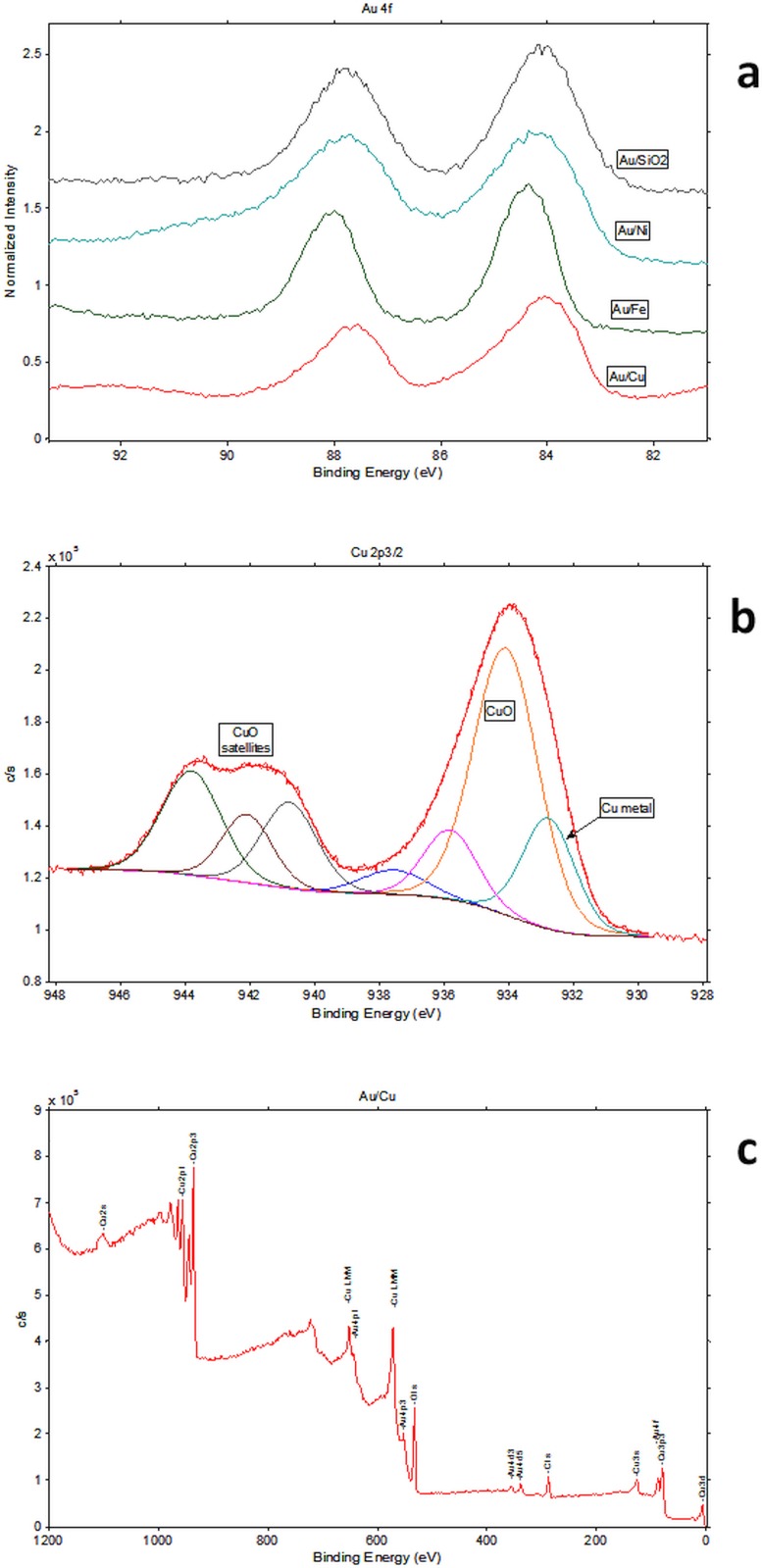
XPS spectra for 1.0% Au/Cu catalyst. Photoemission Au 4f doublet for all Au NP systems (a), Photoemission Cu 2p_3/2_ multiplet with the result of fitting (b) XPS survey spectrum for the Au/Cu system.

The chemical state of Au and supporting material can be estimated from the XPS lines **(Fig 3A)**. For Cu the Cu 2p multiplet has the shape and position characteristic for CuO **(Fig 3B)**. However, the main line of the Cu 2p_3/2_ doublet is very broad and one cannot exclude a metallic component of low intensity **(Fig 3C)**. Such component can indicate a formation of an alloy. The shape of the Au 4f doublet is different than in pure gold and different from other systems studied within the presented work **(Fig 3A)**. The XPS doublet can be fitted by at least 2 sets of lines. One of them has the energy characteristic for pure Au which is close to 84.0 eV for the Au 4f_7/2_ line, while the second one has the higher energy of about 84.5 eV **(Fig 3C)**. Similar position was reported in the NIST database for Au-Cu compounds. Moreover, the composition (Au/X ratio, where X is supporting material) derived from XPS is only slightly higher as for EDXRF indicating to possible alloying with Cu. The part of Cu particles which are not covered by Au oxidize and form CuO on the surface.

The XPS lines of Au 4f level for the Fe supported system have the shape similar to pure Au but the position is shifted to higher binding energy **(S1A Text)**. It can be related with the known effect of increased binding energy for nanoparticles which have very small size of few nanometers. The Fe 2p lines are characteristic for Fe_2_O_3_. Similarly, the chemical state of Ni derived for XPS lines indicates to Ni_2_O_3_ **(S1B Text)**. However, the Au 4f doublet is much broader than for Fe support and it may be an indication for formation of Au-Ni alloys or very small size of Au nanoparticles.

In case of the 1.5% Au/SiO_2_ composite single chemical states of both Au and Si were observed **(S1C Text)**. Binding energy of Au 4f core level is alike in pure Au whereas the position of Si 2p line is characteristic for SiO_2_ compound. The presence of Au and Si in such chemical states would argue in favor location of Au nanoparticles in the pores of larger particles of SiO_2_.

**Fig 4:**
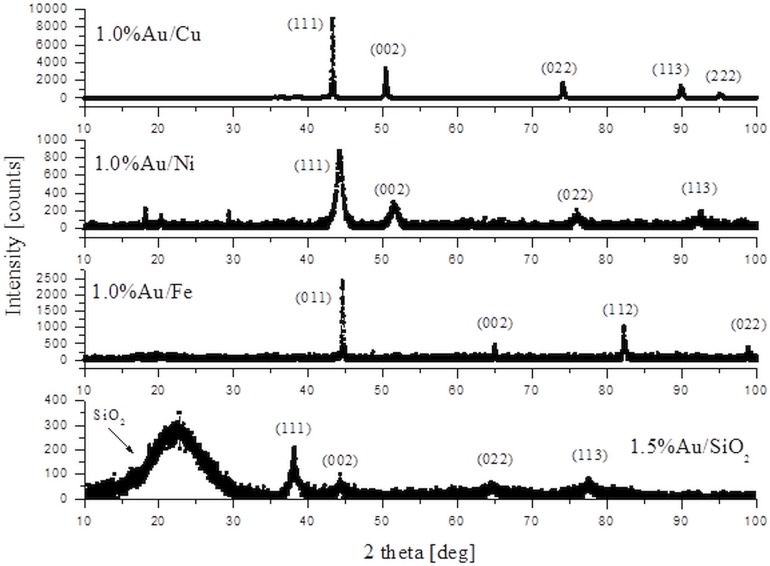
The X-ray diffraction patterns of 1.0%Au/Cu, 1.0%Au/Ni, 1.0%Au/Fe and 1.5%Au/SiO_2_ samples. Miller indices for experimental peaks are marked.

The X-ray diffractograms **(Fig 4)** showed clear peaks for all systems which could be attributed to crystalline supporting material and partly to Au nanoparticles (for the Au/SiO_2_ system). To calculate the lattice parameters of investigated samples the “Chekcell V4” program was used. The values are given as follows: Au/Cu a = 3,618(±0,002)Å, Au/Fe a = 2,869(±0,.006)Å, Au/Ni a = 3,539(±0,016)Å and Au/SiO_2_ a = 4,083(±0,011)Å. The Scherrer equation was used to estimate the average size of crystalline particles. For the Au/Cu and Au/Fe systems the lines coming from the supporting particles were narrow indicating to large particle size which was calculated as 40–50 nm. The results were different for Au/Ni and Au/SiO_2_ samples. The particle size estimated from the Au/Ni most pronounced line was about 2.9 nm. The similar value of 2.8 nm was obtained for the line coming from Au nanoparticles supported by SiO_2_. The part of the diffractogram which could be attributed to silica was a very broad and relatively weak bump at low angle range indicating to rather amorphous form. The X-ray analyses of the samples of the catalysts indicate that the Au NPs in Au/Ni are preserved in a form most similar to the original Au/SiO_2_ form. In these systems, the larger average NPs size was determined by TEM measurements than by X-ray analyses. We are explaining this by a fact that the TEM images of NPs are often ambiguous both due to the superposition of the NPs on the images and their agglomeration. Thus, TEM provides the values higher than the X-ray method. On the other hand, X-ray diffractometers did not indicate crystalline NPs of the 2.9 nm for Au/Cu and Au/Fe which may indicate that the Au crystal structure changed in these materials. However, similarly to Au/Ni we were able to observe by the TEM method the Au NPs of the size below 10 nm. Especially for Au/Cu this seems to comply with the XPS results suggesting the Au/Cu alloying effect (broader XPS lines). In turn, the combination of XPS with X-ray diffractogram data for the Au/Ni, i.e., the original size of Au NPs were preserved on the surface of the Ni carrier (X-ray data) while the broader XPS lines seems to indicate in this case a small Au NPs size.

### B. Glycerol dehydration in the gas phase

**Fig 5:**
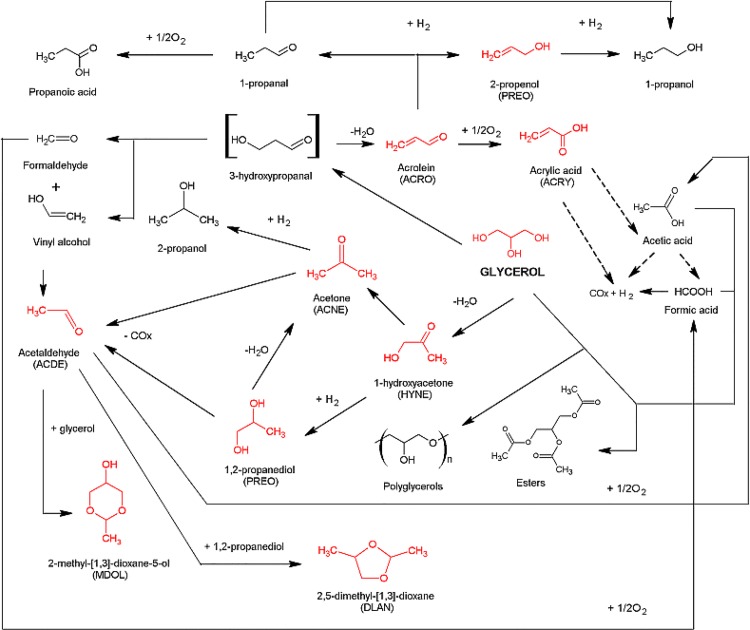
Reaction network during glycerol dehydration. The products that were identified in the current study are marked in red. For the comparison of the identified products with those reported in the literature see review [11], updated by the recent studies [26–31].

Dehydration can be an interesting option for glycerol processing. A small three-carbon glycerol can provide a variety of products. However, similar to oxidation, the products can form a complex mixture of chemical compounds. In **Fig 5,** we briefly illustrated the possible reaction paths that may be observed during glycerol dehydration.

In **Table 3**we present the performance of Au/SiO_2_ vs. SiO_2_ systems in the dehydration of glycerol in the gas phase using 1.5% SiO_2_-supported Au NPs as the catalyst. This was tested under a higher glycerol concentration– 13.6 mol/L (C_3_H_8_O_3_/H_2_O = 99.5/0.5) under an inert gas N_2_ flow of 0.3 mL/min. Interestingly, both systems preferred the formation of hydroxyacetone (HYNE) as the main product. The SiO_2_ catalyzed reaction at 400°C was the only exclusion because 1,2-propanediol (ca. 24%) appeared to dominate HYNE (22%) and was the major product. Conversion was relatively low for both systems, but increased with the increasing temperature to reach a maximal value of ca. 31% for Au/SiO_2_ at 300°C. Other products that were observed were 1,2-propanediol (PRDL), 2,5-dimethyl-[1,3]-dioxane (DLAN), acrolein (ACRO) and acrylic acid (ACRY) (see **Table 3**for the detailed proportions of all of the products.) In comparison to SiO_2_ alone, which provided a relatively low selectivity, Au/SiO_2_ appeared to be more selective, especially at a lower temperature, i.e. at 300°C HYNE selectivity amounted to 55.9% for Au/SiO_2_ **(Table 3 entry 4)** vs. 33.1% for SiO_2_ alone **(Table 3 entry 1).**

**Table 3:** Catalytic performance of SiO_2_-supported Au NPs in undiluted glycerol solutions at 300–400°C^a^.

	Catalyst	Temp. [°C]	TOF^b^	LY^c^	τ_c_ [s]	Conv. [mol%]	Selectivity^d^ [mol%]										HYNE^e^
							ACRO	ACRY	ACNE	ACDE	PRDL	HYNE	PREO	MDOL	DLAN	OS	
1	SiO_2_	300	113.3	99.9	0.0687	4.3	0	0	0	0	22.0	33.1	0	8.1	15.5	21.3	1.4
2		350	113.3	99.8	0.0632	10.8	8.2	8.3	1.6	8.3	19.0	24.8	0	8.3	8.3	13.2	2.7
3		400	113.3	99.6	0.0585	15.2	5.5	5.6	1.1	5.5	23.9	22.2	5.3	12.8	3.7	14.4	3.4
4	1.5% Au/SiO_2_	300	7454.0	99.8	0.0687	4.5	0	0	0	0	7.4	55.9	0	7.6	14.9	14.2	2.5
5		350	7454.0	99.5	0.0632	15.4	5.5	5.5	1.1	5.5	14.9	25.0	11.0	7.7	7.4	16.4	3.9
6		400	7454.0	99.3	0.0585	30.8	4.5	6.7	1.1	6.7	13.5	24.7	6.7	12.6	3.0	20.5	7.6

a/ 13.6 mol/L glycerol (C_3_H_8_O_3_/H_2_O = 99.5/0.5), 200 mg catalyst bed (optionally with 15.0 *μmol* Au), inert gas N_2_ flow 0.3 mL/min

b/ Turnover frequency TOF [h^1^] calculated as TOF = V/n, where V is the molar flow rate of glycerol and n is a number of moles of Au NPs

c/ Liquid phase yield LY [%] experimentally determined total amount of liquid phase flowing out of the reactor

d/ ACRO–acrolein, ACRY–acrylic acid, ACNE–acetone, ACDE–acetaldehyde, PRLD– 1,2-propanediol, HYNE– 1-hydroxyacetone, PREO– 2-propenol, MDOL—2-methyl-[1,3]-dioxane-5-ol, DLAN– 2,5-dimethyl-[1,3]-dioxane, OS–others.

e/ HYNE yield [%].

In **Table 4**we specified the activity of bimetallic systems, where Ni-, Fe- and Cu supported Au NPs, in glycerol dehydration in the gas phase for a glycerol flow of 13.6 mol/L (C_3_H_8_O_3_/H_2_O = 99.5/0.5) with an inert N_2_ gas flow 0.3 mL/min. Similar to SiO_2_ and Au/SiO_2_, HYNE was the main product in this case. Although glycerol conversion on Au/Ni and Au/Fe appeared to be slightly better than those that were observed for the Au/SiO_2_ **(Table 3)**, the Cu supported Au NPs allowed for a significant improvement of conversion and selectivity, i.e. conversion amounted to ca. 63% and HYNE selectivity ca. 70% **(Table 4, entry 1).** The comparison of the reactivity of the carrier metals (Ni, Fe, Cu), which were not supported with Au NPs with those with Au NPs **Table 4**, indicated that bimetallic constructs with Au NPs always improved glycerol conversion. Interestingly, the comparison of the glycerol conversion on the Ni, Fe, Cu supported Au NPs indicates that the Au/Ni, i.e., this system that preserves the original crystal structure of the Au on the SiO_2_ carier (X-ray analyses, **Fig 4**), showed the lowest reactivity at the lowest temperature 300°C **(Table 4, entry 4)**. This seems to prove that interamtellic interactions, in particular, within the Au/Cu system decided the high activity of this catalyst.

**Table 4:** Catalytic performance of Cu-, Ni- and Fe-supported Au NPs in undiluted glycerol solutions at 300–400°C^a^.

	Catalyst	Temp. [°C]	TOF^b^	τ_c_ [s]	LY^c^	Conv. [mol%]	Selectivity^d^ [mol%]										HYNE^e^
							ACRO	ACRY	ACNE	ACDE	PRDL	HYNE	PREO	MDOL	DLAN	OS	
1	1.0% Au/Cu	300	10026.6	0.0357	97.3	63.2^f^	0	0	6.4	0	1.5	70.2	0	0	0	21.9	44.4
2		350	10026.6	0.0328	98.0	54.4	0	0	7.8	0	2.2	56.3	0	1.9	5.0	26.8	30.6
3		400	10026.6	0.0304	99.1	23.9	3.2	6.4	3.8	6.4	15.0	28.7	6.4	12.7	3.2	14.2	6.9
4	1.0% Au/Ni	300	11561.3	0.0371	99.6	8.2	0	0	0	2.3	14.6	39.3	11.2	7.9	7.5	17.2	3.2
5		350	11561.3	0.0341	99.4	21.1	3.7	3.8	0	2.6	17.2	18.7	22.5	9.7	3.7	18.1	3.9
6		400	11561.3	0.0316	99.1	34.7	1.9	3.8	0.6	3.8	15.0	24.4	16.9	13.2	2.5	17.9	8.5
7	1.0% Au/Fe	300	11107.9	0.0412	99.3	11.9	0	0	0	2.2	14.8	44.5	7.4	5.2	7.4	18.5	5.3
8		350	11107.9	0.0379	99.2	27.5	2.6	5.3	0.5	3.4	15.8	22.4	15.8	8.7	3.5	22.0	6.2
9		400	11107.9	0.0351	99.0	43.1	2.6	4.0	0.9	2.6	15.3	23.1	15.8	15.8	3.1	16.8	10.0
10	Cu	400	113.3	0.0304	99.8	7.8	0	0	2.0	3.9	11.7	35.2	0	8.2	11.7	27.3	2.7
11	Ni		113.3	0.0316	99.7	12.4	0	0	1.1	2.3	28.2	28.2	0	9.2	4.9	26.1	3.5
12	Fe		113.3	0.0351	99.5	17.6	4.7	4.7	1,5	4.8	9.4	28.1	9.4	9.4	4.7	23.3	4.9

a/ 13.6 mol/L glycerol (C_3_H_8_O_3_/H_2_O = 99.5/0.5), 200 mg catalyst bed (optionally with 15.0 *μmol* Au), inert gas N_2_ flow 0.3 mL/min

b/ Turnover frequency TOF [h^1^] calculated as TOF = V/n, where V is the molar flow rate of glycerol and n is the moles of Au NPs

c/ Liquid phase yield LY [%] experimentally determined total amount of liquid phase flowing out of the reactor

d/ ACRO–acrolein, ACRY–acrylic acid, ACNE–acetone, ACDE–acetaldehyde, PRLD– 1,2-propanediol, HYNE– 1-hydroxyacetone, PREO– 2-propenol, MDOL– 2-methyl-[1,3]-dioxane-5-ol, DLAN– 2,5-dimethyl-[1,3]-dioxane, OS–others.

e/ HYNE yield [%]

f/ the conversion decreases at 300°C from the original 63,2° to 27,2%, if tested in the second reaction run (reused catalyst).

**Fig 6:**
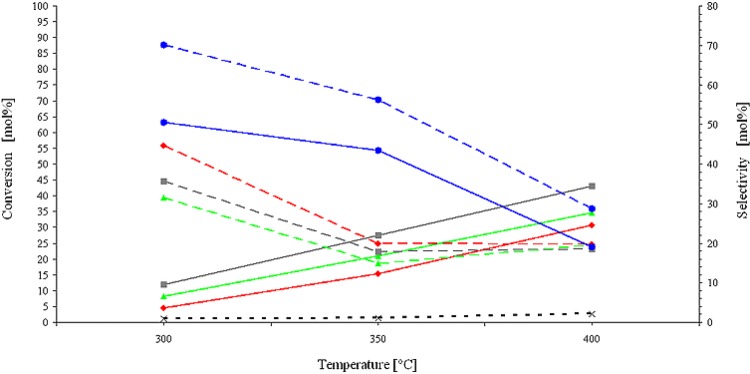
Glycerol conversion (solid lines) and selectivity (dashed lines) vs. temperature, 1.0% Au/Fe (■), 1.0% Au/Ni (▲), 1.0% Au/Cu (●) and 1.5% Au/SiO_2_ (♦). The dotted lines refer to the yield of 1-hydroxyacetone; black dotted line (**x**)–the total conversion of the blank sample, solid lines describe glycerol conversion. Reaction conditions: 200 mg of catalyst (10.0–15.0 μmol Au), 300–400°C, glycerol concentration 13.6 mol/L and nitrogen flow 0.3 mL/min.

In **Fig 6**we illustrate the influence of temperature on conversion and selectivity for the catalysts that were investigated. Paradoxically, unlike all other catalytic systems, both the conversion and HYNE selectivity decreases with an increase in temperature for the Au/Cu bimetallics. This paradox can be explained by the high reactivity of the the Cu supported Au NPs. The higher the temperature is the higher the reactivity and the lower the selectivity of the catalyst is. At the same time, undesired high molecular products of glycerol condensation are also formed with higher yields, thus deactivating the catalyst, which results in a lower conversion (ca. 63% at 300°C vs. 24% at 400°C).

In **Table 5**we illustrate the influence of the glycerol concentration in the flow gases. Thus, we diluted streaming glycerol with water vapor to provide a flow of 1.0 mol/L glycerol (C_3_H_8_O_3_/H_2_O = 9/91; N_2_ flow of 0.3 mL/min). As previously, among the tested sytems SiO_2_, Au/SiO_2_, Cu alone and Au/Cu, the latter appeared the most active providing almost 72% HYNE selectivity at 250°C, however, conversion here was lower ca. 24%. Similar to water-free flow a paradoxical conversion (selectivity) vs. temperature behavior was also observed.

**Table 5:** Catalytic performance of Cu- and SiO_2_-supported Au NPs in diluted glycerol solutions at 250–400°C^a^.

	Catalyst	Temp. [°C]	TOF^b^	τ_c_ [s]	LY^c^	Conv. [mol%]	Selectivity^d^ [mol%]								HYNE^e^
							ACRO	ACRY	ACNE	ACDE	PRDL	HYNE	MDOL	OS	
1	SiO_2_	250	10.4	0.0752	99.9	2.9	0	0	7.3	0	12.1	55.0	0	25.6	1.6
2		370	10.4	0.0612	99.9	3.8	0	0	22.2	0	6.7	46.8	0	24.3	1.8
3		400	10.4	0.0584	99.9	4.3	0	0	25.0	0	0	50.0	0	25.0	2.2
4	1.5% Au/SiO_2_	250	686.4	0.0752	99.8	4.1	0	0	7.7	0	7.0	69.9	0	15.4	2.9
5		370	686.4	0.0612	99.6	6.3	0	0	7.4	0	10.3	54.6	10.3	17.4	3.4
6		400	686.4	0.0584	99.4	15.9	5.3	10.5	2.6	5.3	6.9	33.2	14.2	22.0	5.3
7	Cu	250	10.4	0.0391	98.9	22.7	0	0	7.5	0	6.8	45.9	0	39.8	10.4
8		370	10.4	0.0318	99.3	12.3	0	0	10.7	0	7.1	53.6	0	28.6	6.6
9		400	10.4	0.0304	99.0	2.7	0	0	12.0	0	10.9	36.4	10.9	29.8	1.0
10	1.0% Au/Cu	250	923.3	0.0391	98.1	26.2	0	0	3.7	0	2.0	71.8	2.8	19.8	18.8
11		370	923.3	0.0318	98.8	16.3	0	0	5.1	0	1.5	56.4	3.6	33.3	9.2
12		400	923.3	0.0304	99.8	5.4	0	0	8.7	0	5.3	35.1	12.3	38.6	1.9

a/ 1.0 mol/L glycerol (C_3_H_8_O_3_/H_2_O = 9/91), 200 mg catalyst bed (optionally with 10.0–15.0 *μmol* Au), inert gas N_2_ flow 0.3 mL/min

b/ Turnover frequency TOF calculated as TOF = V/n, where V is the molar flow rate of glycerol and n is the moles of Au NPs

c/ Experimentally determined total amount of liquid phase flowing out of the reactor

d/ ACRO–acrolein, ACRY–acrylic acid, ACNE–acetone, ACDE–acetaldehyde, PRLD– 1,2-propanediol, HYNE– 1-hydroxyacetone, PREO– 2-propenol, MDOL– 2-methyl-[1,3]-dioxane-5-ol, DLAN– 2,5-dimethyl-[1,3]-dioxane, OS–others.

e/ HYNE yield [%]

It is interesting to compare our results with those that have been reported recently for the so-called glycerol catalytic reactive distillation in the liquid phase at a temperature of 240°C and a reduced pressure of 98 kP [32]. The best results were reported for a copper-chromite catalyst in a batch mode that amounted to a conversion value of ca. 64% and a selectivity of ca. 67% (after the recalculation of the reported values to include unconverted glycerol residues, in order to comply with the full mass balance performed in the current studies). This means that in our experiments in the gas phase, the activation of Cu using Au NPs produces results that are similar to those that are obtained for catalytic reactive glycerol distillation in the liquid phase on copper-chromite catalysts (conversion ca. 63%, HYNE selectivity ca. 70%).

## Conclusions

Au NPs have recently been used in a variety of reactions. In this study, we investigated different metal pairings of Au NPs as potential catalysts for glycerol dehydration for the first time. All of the systems preferred the formation of hydroxyacetone (HYNE). Although the bimetallics that were tested Au/(Ni, Fe, Cu) appeared to be more active than the Au/SiO_2_ system, only Cu supported Au NPs appeared to produce a high conversion (ca. 63%) and selectivity (ca. 70%). In summary, Cu-supported Au NPs can form an interesting catalytic system for glycerol dehydration as the prefer the formation of hydroxyacetone in both the undiluted and water diluted gas phases.

## Supporting Information

S1 Table The chemical composition of the Au/Ni catalyst (EDS). (DOC)

S2 Table Au content as determined by EDXRF and XPS analyses. (DOC)

S1 Fig Representative SEM and TEM images of the Au/Ni catalyst. SEM (a) and TEM (b,c) images of 1.0% Au/Ni catalyst showing Au NPs (c) mainly in the form of Au conglomerates in the amorphous-nanocrystalline Si and Ni matrix and the electron diffraction pattern from these areas (b). Particle size distribution of Au NPs (d). The chemical compositions in these areas are presented in S1 Table.
(TIF)

S2 Fig Representative SEM and TEM images of the Au/Fe catalyst. SEM and TEM images of the Au/Fe catalyst: a—SEM image, b, c—TEM bight and dark field images of the aggregates of Au and CaO nanoparticles, d—HRTEM image of the Au NPs. In the corner of b image the electron diffraction pattern from Au and CaO phases is situated.
(TIF)

S1 Text XPS spectra for 1.0% Au/Fe (a), Au/Ni (b) and Au/SiO_2_ (c) catalysts. (DOCX)
