# Supplementary material for: Catalytic Gas-Phase Glycerol Processing over SiO2-, Cu-, Ni- and Fe- Supported Au Nanoparticles
Source: PLoS One. 2015 Nov 18;10(11):e0142668. doi: 10.1371/journal.pone.0142668 (PMC4651318; doi:10.1371/journal.pone.0142668)
Supplement: S1 Table — (DOC) [file pone.0142668.s001.doc]

**S1 Table** **The chemical composition of the Au/Ni catalyst (EDS).**

| Chemical element | C [%at.] | D [%at.] | E [%at.] | F [%at.] | G [%at.] |
| --- | --- | --- | --- | --- | --- |
| O | - | 10.9 | - | - | 20.0 |
| Ni | 9.2 | 9.0 | 65.3 | 58.7 | 56.9 |
| Au | 80.2 | 69.6 | - | 1.7 | 1.0 |
| Si | 10.6 | 10.5 | 34.7 | 39.6 | 22.1 |
