# Supplementary material for: Catalytic Gas-Phase Glycerol Processing over SiO2-, Cu-, Ni- and Fe- Supported Au Nanoparticles
Source: PLoS One. 2015 Nov 18;10(11):e0142668. doi: 10.1371/journal.pone.0142668 (PMC4651318; doi:10.1371/journal.pone.0142668)
Supplement: S2 Table — (DOC) [file pone.0142668.s002.doc]

**S2 Table** **Au content as determined by EDXRF and XPS analyses.**

|  | Catalyst | Au concentration, % (m/m) | |
| --- | --- | --- | --- |
|  |  | EDXRF | XPS |
| 1 | 1.5% Au/SiO_2_ | 1.52 ± 0.12 | 3.1± 0.1 |
| 2 | 1.0% Au/Cu | 1.18 ± 0.051 | 9.6 ± 0.1 |
| 3 | 1.0% Au/Ni | 1.11 ± 0.030 | 50.2 ± 0.1 |
| 4 | 1.0% Au/Fe | 1.02 ± 0.042 | 21.6± 0.1 |
