# Supplementary figures and images for: Catalytic Gas-Phase Glycerol Processing over SiO2-, Cu-, Ni- and Fe- Supported Au Nanoparticles
Source: PLoS One. 2015 Nov 18;10(11):e0142668. doi: 10.1371/journal.pone.0142668 (PMC4651318; doi:10.1371/journal.pone.0142668)

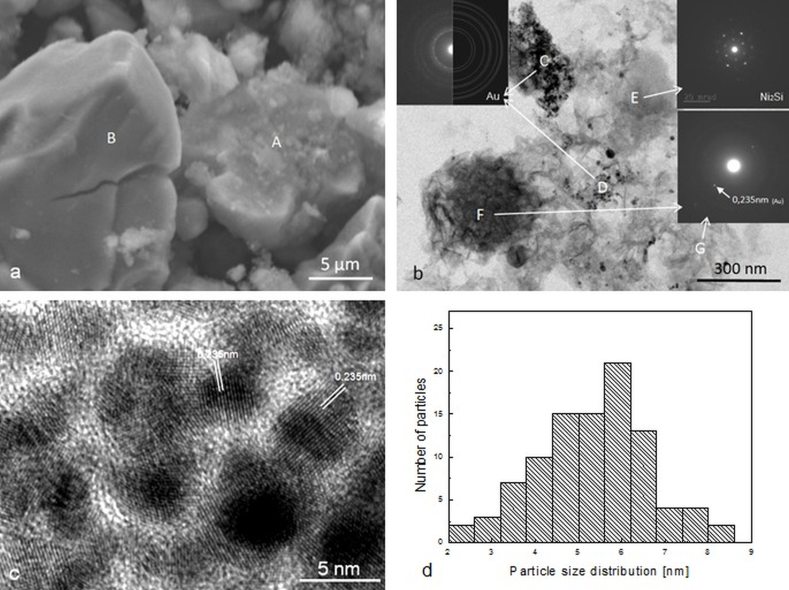

Supplement: S1 Fig — SEM (a) and TEM (b,c) images of 1.0% Au/Ni catalyst showing Au NPs (c) mainly in the form of Au conglomerates in the amorphous-nanocrystalline Si and Ni matrix and the electron diffraction pattern from these areas (b). Particle size distribution of Au NPs (d). The chemical compositions in these areas are presented in S1 Table. (TIF) [file pone.0142668.s003.tif]

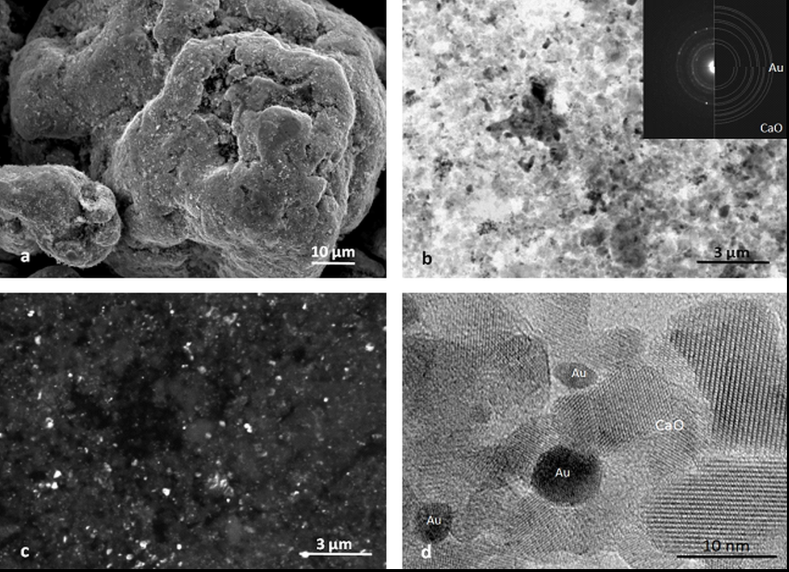

Supplement: S2 Fig — SEM and TEM images of the Au/Fe catalyst: a—SEM image, b, c—TEM bight and dark field images of the aggregates of Au and CaO nanoparticles, d—HRTEM image of the Au NPs. In the corner of b image the electron diffraction pattern from Au and CaO phases is situated. (TIF) [file pone.0142668.s004.tif]
