# Supplementary material for: Catalytic Gas-Phase Glycerol Processing over SiO2-, Cu-, Ni- and Fe- Supported Au Nanoparticles
Source: PLoS One. 2015 Nov 18;10(11):e0142668. doi: 10.1371/journal.pone.0142668 (PMC4651318; doi:10.1371/journal.pone.0142668)
Supplement: S1 Text — (DOCX) [file pone.0142668.s005.docx]

**S1 Text** XPS spectra for 1.0% Au/Fe, Au/Ni and Au/SiO_2_ catalysts.

1. 1.0% Au/Fe

1. 1.0 % Au/Ni

(c) 1.5% Au/SiO_2_
